# Unveiling the Nature of Chemical Bonds in Real Space

**DOI:** 10.1021/jacs.4c05673

**Published:** 2024-07-18

**Authors:** Takeshi Hara, Masatoshi Hasebe, Takao Tsuneda, Toshio Naito, Yuiga Nakamura, Naoyuki Katayama, Tetsuya Taketsugu, Hiroshi Sawa

**Affiliations:** † Department of Applied Physics, Nagoya University, Nagoya 464-8603, Japan; ‡ Graduate School of Chemical Sciences and Engineering, Hokkaido University, Sapporo 060-8628, Japan; § Department of Chemistry, Faculty of Science, Hokkaido University, Sapporo 060-0810, Japan; ∥ Graduate School of System Informatics, Kobe University, Kobe 657-0013, Japan; ⊥ Graduate School of Science and Engineering, Ehime University, Matsuyama 790-8577, Japan; # Japan Synchrotron Radiation Research Institute (JASRI), SPring-8, Hyogo 679-5198, Japan; ∇ Institute for Chemical Reaction Design and Discovery (WPI-ICReDD), Hokkaido University, Sapporo 001-0021, Japan

## Abstract

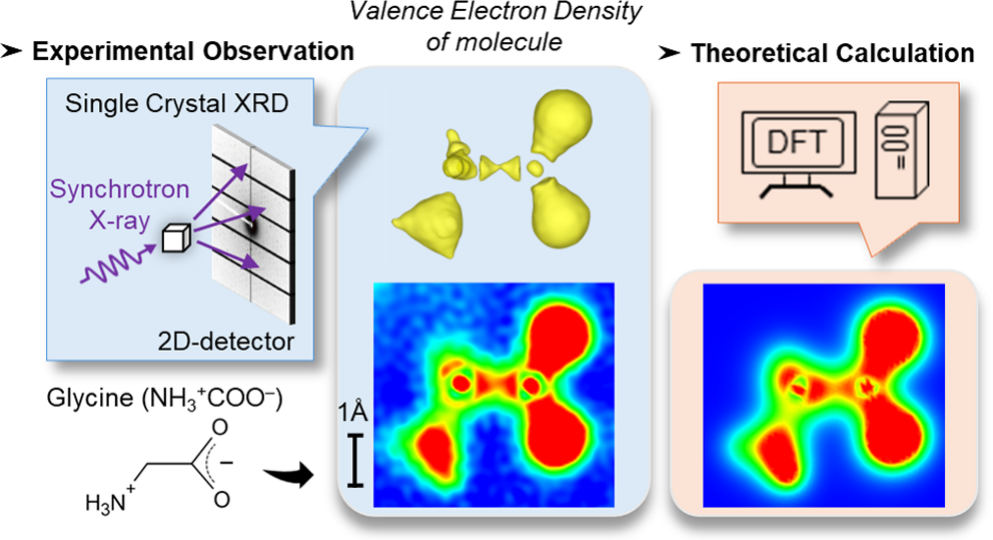

Recent advent of diverse chemical entities necessitates a re-evaluation of chemical bond concepts, underscoring the importance of experimental evidence. Our prior study introduced a general methodology, termed Core Differential Fourier Synthesis (CDFS), for mapping the distribution of valence electron density (VED) in crystalline substances within real space. In this study, we directly compare the VED distributions obtained through CDFS with those derived from high-accuracy theoretical calculation using long-range corrected density functional theory, which quantitatively reproduces accurate orbital energies. This comparison serves to demonstrate the precision of the CDFS in replicating complex details. The VED patterns observed experimentally exhibited detailed structures and phases of wave functions indicative of sp^3^ hybrid orbitals, closely aligning with theoretical predictions. This alignment underscores the utility of our approach in gathering quantum chemical data experimentally, a crucial step for discussing the chemical properties, such as reaction mechanisms.

## Introduction

Recent progress in organic synthesis has led to the discovery of a wide variety of recombinant chemical species. Alongside these innovations, novel methodologies have been instrumental in uncovering their unique structures and functionalities. This shift has prompted a re-evaluation of chemical bond theories, moving beyond simplified hybridized orbitals.^1−8^ Experimental efforts to understand chemical bonds have expanded, employing techniques such as infrared spectroscopy,^9^ X-ray photoelectron spectroscopy,^10^ soft X-rays emission spectroscopy,^11^ Compton scattering,^12−15^ and high harmonics.^16^ These techniques probe the electronic states of organic compounds in momentum space, though exploring these states in real space poses significant challenges. In response, we introduced the Core Differential Fourier Synthesis (CDFS) method, which extracts the valence electron density distribution (VED) within crystals from single crystal X-ray diffraction data using synchrotron radiation.^17−20^ This approach bypasses the reliance on quantum mechanical models for the electronic states. CDFS allows for the observation of the phase relationships and detailed structure of hybridized orbitals at the atomic level, facilitating direct comparisons with quantum chemical predictions. To support such analyses, long-range corrected density functional theory (LC-DFT) emerges as the most suitable computational method, which can quantitatively reproduce molecular valence orbital energies.^21^ This enables a detailed alignment with experimental VED findings. Our findings clearly show which wave functions contribute to the bond formation and how and can be directly compared with highly accurate DFT calculations. Therefore, experimental observation of VED could contribute to the development of functional materials, where electronic states within molecules are linked to physical and chemical properties.

In this study, we have confirmed that CDFS enables the visualization of electron distribution in organic crystalline materials with wave function-level accuracy. We conducted comparative analyses of the VEDs in molecular crystals, employing both the CDFS method and LC-DFT. For this investigation, we selected two typical molecular crystals, Glycine and Cytidine, as representative samples. These samples are historically well-known, and their crystal structures were previously determined by diffraction methods.^22−26^

## Results and Discussion

**Figure 1:**
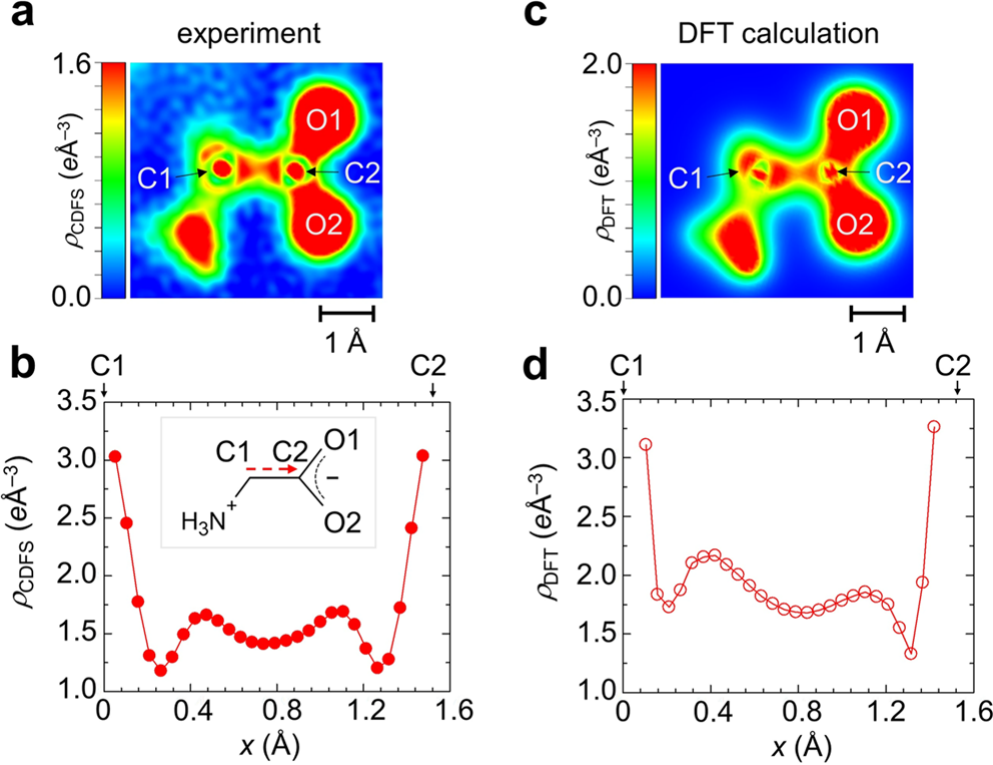
Experimental and theoretical VEDs of the Glycine molecule. (a) 2D contour plot and (b) 1D curve plot of the experimental VED, ρ_CDFS_(***r***), along the C1–C2 axis in Glycine. (c) 2D contour plot and (d) 1D curve plot of the theoretical VED, ρ_DFT_(***r***), focusing on the C1–C2 bond. These contour and curve plots are generated from 3D VED data using the VESTA software.^27^

First, we focus on the experimental observation of the carbon–carbon σ-bond. Figure 1a displays a two-dimensional (2D) electron density contour map showcasing the experimental VED ρ_CDFS_(***r***) for Glycine molecules in their crystalline form (additional visualizations, including a 3D surface plot and a contour map of the experimental VED of Glycine, are available in Movie S1). Moving beyond the simplified depictions commonly found in educational materials, we observe a notably uneven VED distribution throughout the molecule. Specifically, in the vicinity of carbon atoms C1 and C2, we identify clear nodal planes near each atom with a pronounced decrease in electron density at the midpoint of the C1–C2 bond. To ascertain whether these intricate patterns are intrinsic characteristics, we investigated a theoretical model depicting electron density distribution along this bond, using hybrid orbitals derived from the 2s and 2p orbitals of an isolated carbon atom (detailed in Section S2). At a distance of approximately 0.2 Å from the carbon atom, a nodal plane consistent with our empirical findings emerges within the hybrid orbital distribution ϕ_C1_(***r***), as illustrated in Figure S5. This observation confirms the presence of the nodal plane of the 2s orbital in the real VED. It is important to note here that employing high-energy X-ray techniques is essential for a deeper understanding of the chemical bond nature, as they offer the spatial resolution needed to discern nodal structures resulting from orbital hybridization, as discussed in Section S3. By experimentally discerning even the structural features of the wave functions in this manner, it becomes feasible to elucidate which wave functions contribute to bond formation and how.

Subsequently, we conducted LC-DFT calculations to compare with the experimentally observed VED of Glycine molecules. It is particularly noteworthy that this calculation is based on a single molecular structure experimentally obtained without structural optimization using high-precision crystal coordinates derived from high-angle X-ray analysis. Here, the theoretical VED, ρ_DFT_(***r***), is derived from the occupied molecular orbitals, excluding the 1s^2^ core electrons of the C, N, and O atoms. Fundamentally, the C(sp^3^)–C(sp^3^) bond is represented by the bonding orbitals, Ψ(***r***) = *C*(ϕ_C1_(***r***) + ϕ_C2_(***r***)), and the distribution of |Ψ(***r***)|^2^ is depicted in Figure S5. However, in contrast with |Ψ(***r***)|^2^, the theoretical VED (Figure 1c,1d) shows a deeper dip at the center of the C1–C2 bond. This variance stems from the presence of nodal planes within the occupied molecular orbitals in the theoretical VED. The theoretical electron density, ρ_DFT_(***r***), along the C1–C2 bond is about 10% greater than that of ρ_CDFS_(***r***), a discrepancy mainly due to different approaches in incorporating temperature effects between the experimental and theoretical VEDs, as discussed in Section S4. Furthermore, there are two peaks of the VEDs on either side of the dip in the center of the C1–C2 bond, but their heights are different in theory, whereas experimentally they are almost the same height (Figure 1b). This effect is attributed to the polarization of Glycine molecules in their solid form, where the uneven charge distribution around the NH_3_^+^ and COO^–^ groups leads to the uniform electronic distribution along the C1–C2 bond. The experimental VED is likely influenced by the crystal field effects, resulting in a more uniform electron density distribution along the C1–C2 bond. Crystal field effects further contribute to the differentiation of the C–H bonds, as elaborated in Section S6. It is important to note that the theoretical analysis was based on an isolated Glycine molecule in its zwitterionic (NH_3_CHCO_2_) form, focusing on the energy minimum state. To more accurately assess the consistency of experimental ρ_CDFS_(***r***) and theoretical ρ_DFT_(***r***), it would be more appropriate to perform calculations using a model that includes intermolecular interactions with a crystal structure.

We also focus on the carbon–oxygen bonds within the Glycine molecule, specifically the C2–O1 and C2–O2 bonds, which participate in chemical resonance. Figure S13a depicts a 1D plot of ρ_CDFS_(***r***) along these bonds. The electron density profiles of the C2–O1 and C2–O2 bonds diverge significantly from that observed in the C1–C2 bond, as depicted in Figure 1, indicative of the differences in electronegativity between oxygen and carbon. Despite this, the bond lengths and VED profiles of the C2–O1 and C2–O2 bonds are remarkably similar, as confirmed by the theoretical VED, ρ_DFT_(***r***), shown in Figure S13b. This similarity suggests electron delocalization between O1 and O2, leading to virtually identical C2–O1 and C2–O2 bonds via resonance, aligning with the traditional understanding of stabilization in carboxylate ions through resonance.

**Figure 2:**
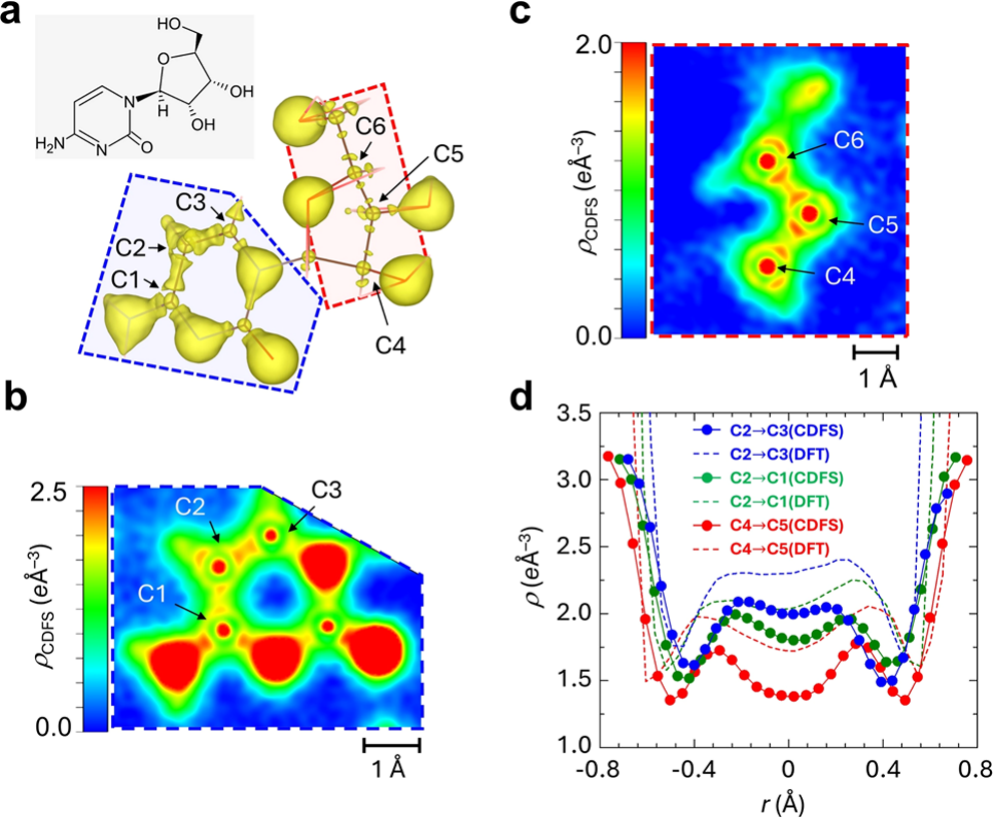
Experimental VED of the Cytidine molecule. (a) 3D surface plot of experimental VED, ρ_CDFS_(***r***), for the Cytidine molecule at an isosurface threshold of 1.7 *e*/Å^3^. The bond lengths of C2–C1, C2–C3, and C4–C5 bonds are 1.42967(18), 1.35766(19), 1.52701(15) Å, respectively, alongside Wiberg bond indices^28^ of 1.16 (C1–C2), 1.61 (C2–C3), and 0.97 (C4–C5). (b) 2D contour plots of ρ_CDFS_(***r***) in the six-membered ring and (c) those in the five-membered ring. (d) 1D curve plots of experimental VED, ρ_CDFS_(***r***), (solid lines) and theoretical VED, ρ_DFT_(***r***), (dashed lines) across C2 → C1(green), C2 → C3(blue), and C4 → C5(red) bonds. The horizontal axis is normalized to each bond length with *r* = 0 denoting the midpoint of the bond.

Moreover, we explore the VED variations across bonds of different orders, such as single and double bonds, using the Cytidine molecule as an example. Figure 2 presents the 3D surface and 2D contour maps of VED, ρ_CDFS_(***r***), of Cytidine in its solid form (with 3D surface plots of both experimental and theoretical VEDs of Cytidine available in Movie S2). Analyzing C2=C1 and C2=C3 bonds in the six-membered and C4=C5 bonds in the five-membered rings, we observe distinct electron density distributions correlated with the bond orders. The 1D plots for these bonds in Figure 2d reveal a clear pattern: electron density increases and bond length decreases with higher bond orders, a trend that is consistent with the theoretical VED, ρ_DFT_(***r***).

**Figure 3:**
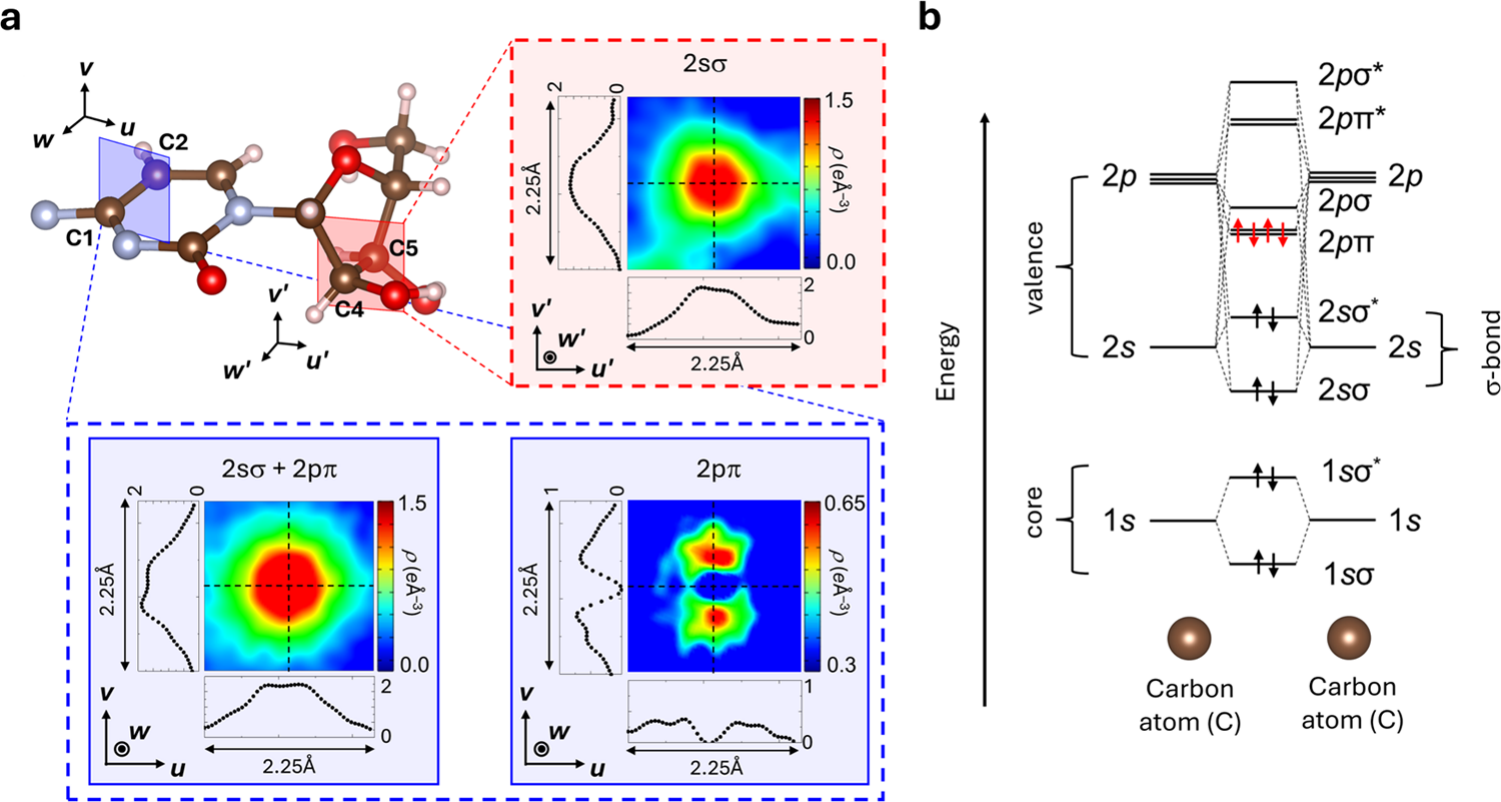
Visualization of π-orbital through the combination of experimental VED and theoretical calculations. (a) Cross section of the C4–C5 bond in experimental VED ρ_CDFS_(***r***) is shown within the red dashed frame, and the cross sections of the C2–C1 bond in ρ_CDFS_(***r***) (left side) and ρ_CDFS_(***r***) – ρ_DFT,2σ_(***r***) (right side) are shown in the blue dashed frame. (b) Energy level representation for a C=C double bond, illustrating the distinct energy levels of two carbon atoms.

In particular, there is a significant difference in the height of the experimentally observed VED between the C–C bonds within the six-membered ring and those within the five-membered ring. This can be understood as the presence or absence of the contribution of π-bonds formed by the 2p orbitals of carbon atoms. Here, we elucidate the method for extracting electron densities of specific molecular orbitals from experimental VEDs, highlighting the crystal field effect and molecular reactivity. It is well established that in VEDs, the π-bond possesses higher energy levels than σ-bonds, as illustrated in Figure 3b. This raises the question of whether we can pinpoint the distribution of π-electron density within the molecule. Here, we focus on the C2–C3 bond in the six-membered ring, which includes a π-bond component, and the C4–C5 bond in the five-membered ring of the Cytidine molecule, which lacks a π-bond component. As shown in Figure 3a, the ρ_CDFS_(***r***) in the cross section of the C4–C5 bond reveals isotropic distribution, consistent with a σ-bond. Similarly, the cross section of the C2–C1 bond within the six-membered ring associated with π-orbitals also exhibits a nearly isotropic pattern due to the overlapping of σ- and π-bonds, complicating their spatial resolution. However, through LC-DFT calculations, we have managed to distinguish between them (further details in Section S7). The energy diagram for an isolated C=C bond, depicted in Figure 3b, clarifies the energetic distinction between the σ- and π-bonds. The concordance between ρ_CDFS_(***r***) and ρ_DFT_(***r***) supports the notion that theoretical σ-orbital densities can accurately represent experimental ones. Consequently, we adopted ρ_DFT,2σ_(***r***) to denote the electron density distribution associated with the 2σ bond, derived from theoretical calculations. The outcome of subtracting ρ_DFT,2σ_(***r***) from ρ_CDFS_(***r***) is illustrated in Figure 3a, revealing a distribution with a nodal plane along and perpendicular to the bond axis, characteristic of π-orbitals. This indicates that the actual VED distribution on the molecule aligns with the molecular orbitals. The theoretical ρ_DFT_(***r***) enables the identification of higher-energy orbitals within the experimental ρ_CDFS_(***r***), which are located in positions nearly identical with those of other molecular orbitals and are influenced by crystal and external fields. The experimental visualization of the electronic distribution of π-bonds within molecules in this manner can contribute to various research fields where π–π interactions influence their functions and structural stability, such as organic semiconductors^29^ and DNA double helix.^30^

**Figure 4:**
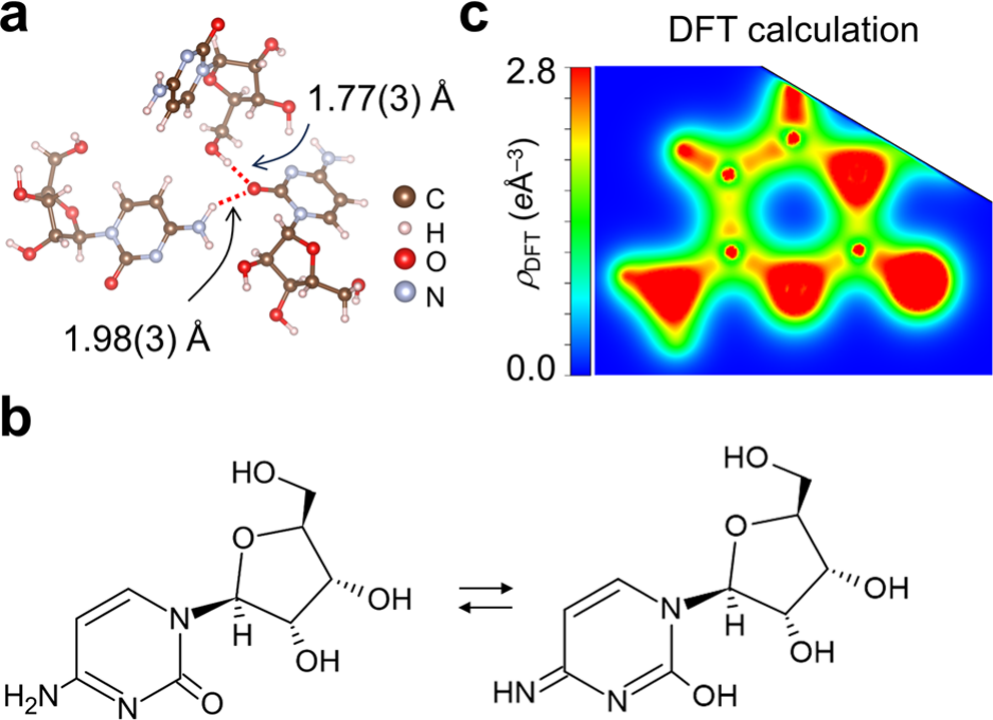
(a) Crystallographic coordination environment around the oxygen atom bonded to the hetero ring. (b) The chemical resonance in the hetero ring is facilitated by proton exchange with adjacent molecules. (c) 2D contour plots of the theoretically calculated ρ_DFT_(***r***) in the six-membered ring, corresponding to the experimental one shown in Figure 2b.

In the molecular structure of Cytidine, C–N and C=N bonds as well as C–C and C=C bonds are present in the six-membered ring. However, as shown in Figures 2b and 4c, the bonds between carbon and nitrogen within the hetero ring and the amino group exhibit similar distributions in both the experimental and theoretical VEDs. This similarity is likely due to the chemical resonance of the hetero ring induced by hydrogen exchange (Figure 4). In the crystal, the oxygen atom bonded to the hetero ring is close to the OH of the carbonyl group and the NH_2_ of the amino group of adjacent molecules (Figure 4a). This proximity is predicted to facilitate hydrogen exchange between them, inducing the rearrangement of C–N and C=N bonds and smoothing out differences (Figure 4b). While such hydrogen exchange is unstable in isolated molecules, it appears to contribute to stabilization in the crystal form. The experimentally obtained VED, which is an average over time and space, accurately replicates this predicted behavior. We demonstrated that by using high-precision experimental structural parameters obtained from high-angle analysis, the theoretical VED can also reproduce the chemical resonance within the heterocyclic ring based on intermolecular interactions within the crystal (Figure 4c). Thus, the combination of experimental VED and theoretical calculations provides a comprehensive understanding of the electronic states of molecules in the crystal.

## Conclusions

In conclusion, we experimentally revealed real-space electron distributions of chemical bonds. The experimental VEDs provide comprehensive quantitative insights into the real nature of chemical bonds, including the chemical resonance observed in the conjugated systems of the carboxylgroups. Furthermore, we discovered that experimental VEDs could accurately extract the precise shapes of specific chemical bonds, such as 2pπ-bonds, even amidst spatial overlap with other orbitals in real space. This methodology enables us to visualize the transformations of real-space chemical bonds that occur during chemical reactions, thereby contributing to the full understanding of chemical bonds and chemical reactions.

## Materials and Methods

### Sample Synthesis

Polycrystalline α-Glycine was purchased (for electrophoresis, >99.0%, Tokyo Chemical Industry Co., Ltd.) and its single crystals were prepared by recrystallization from hot aqueous solution. d-Cytidine, synthesized using the same method as described in a previous study,^31^ was kindly supplied by Dr. G. Ueno (RIKEN) and Prof. K. Sugimoto (Kindai University).

### Single Crystal XRD Experiments and Structure Analysis

Single crystal XRD experiments were conducted at BL02B1 beamline in SPring-8,^32^ using the quarter-circle diffractometer (Rigaku Co., Japan), with diffraction reflections detected by a two-dimensional semiconductor detector, PILATUS3 X CdTe (DECTRIS Ltd., Switzerland). X-ray energies of 40 and 37 keV were used for Glycine and Cytidine, respectively. Temperature variation was achieved using a helium-gas-blowing device (Japan Thermal Engineering Co., Ltd., Japan). The intensities of Bragg reflections were collected by CrysAlisPro program.^33^ Diffraction reflection averaging and crystal structure analysis were performed using SORTAV program^34^ and JANA2006 program,^35^ respectively. VESTA program^27^ was employed for drawing the crystal structure and electron density.

The structural analyses of Glycine and Cytidine were performed at 45 and 35 K, respectively. In the present XRD experiments, the resolution limit of Glycine was *d*_min_ = 0.28 Å [(sin θ/λ)_max_ = 1.786 Å^–1^] and that of Cytidine was *d*_min_ = 0.30 Å [(sin θ/λ)_max_ = 1.6565 Å^–1^]. Accurate determination of structure factor *F*_cal_(***K***) is crucial for the VED analysis using the CDFS method to obtain phase terms of crystal structure factors. To achieve this requirement, we refined the structure parameters using only high-angle data, a technique known as “high-angle refinement” (Section S1).

### VED Analysis Using the CDFS Method

The CDFS method, a technique for directly observing a 3D VED in crystals, employs single crystal X-ray diffraction data obtained from synchrotron radiation. A detailed explanation can be found in previous papers.^17−20^ In this method, the experimental VED distribution is derived by subtracting the calculated core electron density from the experimental total electron density. In both cases, Glycine (C_2_H_3_NO_3_) and Cytidine (C_5_H_9_N_2_O_3_), the core electron density corresponds to 1s^2^ orbital density of C, N, and O atoms. Therefore, the phase relationship and distribution of the hybridized orbitals by 2s and 2p should be visible.

### DFT Calculation

Theoretical valence orbital calculations were performed using DFT with LC-BLYP functional (μ = 0.47)^36^ and cc-pVTZ basis set by Gaussian 16 Revision A.03 program.^37^ The structure parameters determined in the present XRD experiments were used for the calculations (Tables S2 and S5). The structures, orbitals, and electron densities are illustrated using ChemCraft program, while the contour plots are drawn using the VESTA program.^27^

## Supporting Information Available

Additional analytical information on experimental and theoretical data. (PDF)
Movie S1: This movie demonstrates the three-dimensional (3D) surface plot and an animation of the two-dimensional (2D) tomographic image of experimental valence electron density of a Glycine molecule in a crystal. (MPG)
Movie S2: This movie demonstrates an animation of the three-dimensional (3D) surface plots of experimental valence electron density and theoretical valence electron density of a Cytidine molecule in a crystal. (MPG)

## Supplementary Material

ja4c05673_si_001.pdf

ja4c05673_si_002.mpg

ja4c05673_si_003.mpg
