# Supplementary material for: Unveiling the Nature of Chemical Bonds in Real Space
Source: J Am Chem Soc. 2024 Jul 18;146(34):23825–30. doi: 10.1021/jacs.4c05673 (PMC11363912; doi:10.1021/jacs.4c05673)
Supplement: Supplementary file 1 — ja4c05673_si_001.pdf [file ja4c05673_si_001.pdf]

Supporting Information

Unveiling the Nature of Chemical Bonds in Real-Space

Takeshi Hara[1], Masatoshi Hasebe[2], Takao Tsuneda[3,4], Toshio Naito[5],
Yuiga Nakamura[6], Naoyuki Katayama[1], Tetsuya Taketsugu[3,7], and Hiroshi Sawa[1*]

[1]*Department of Applied Physics, Nagoya University, Nagoya 464-8603, Japan*

[2]*Graduate School of Chemical Sciences and Engineering, Hokkaido University, Sapporo 060-8628, Japan*

[3]*Department of Chemistry, Faculty of Science, Hokkaido University, Sapporo 060-0810, Japan*

[4] *Graduate School of System Informatics, Kobe University, Kobe 657-0013, Japan*

[5]*Graduate School of Science and Engineering, Ehime University, Matsuyama 790-8577, Japan*

[6]*Japan Synchrotron Radiation Research Institute (JASRI), SPring-8, Hyogo 679-5198, Japan*

[7]*Institute for Chemical Reaction Design and Discovery (WPI-ICReDD), Hokkaido University, Sapporo 001-0021, Japan*

* Corresponding author: Hiroshi Sawa
Email: hiroshi.sawa@cc.nagoya-u.ac.jp

Contents

Supporting Section 1 | Structure analysis through high-angle refinement

Supporting Section 2 | Straightforward model for describing the covalent bond derived from sp hybridized orbitals composed of 2s and 2p orbitals

Supporting Section 3 | DFT calculation

Supporting Section 4 | Effects of spatial resolution and temperature on VED

Supporting Section 5 | The quantitative differences between $\rho_{\mathrm{CDFS}}(\boldsymbol{r})$ and $\rho_{\mathrm{DFT}}(\boldsymbol{r})$

Supporting Section 6 | The experimental VEDs of C-O, C-N and C-H bonds in a Glycine molecule

Supporting Section 7 | Visualizing the distribution of σ-bond from experimental VEDs subtracting the theoretical distribution of π-bond

Supporting References

Supporting Section 1 | Structure analysis through high-angle refinement

In the realm of structural analysis, a long-standing challenge has been the inherent difficulty in distinguishing the anisotropy arising from atomic thermal vibrations from that attributed to bonding electrons [S1-S5]. This intricacy often hinders the attainment of precise structural parameters. To avoid this issue, a technique known as "high-angle analysis" has been proposed [S6-S9] and applied.

Figure S1a illustrates the $Q = \sin\theta/\lambda$ dependency of the total, core, and valence electrons within the atomic scattering factor of carbon. It is noteworthy that in high Q region, the contributions of core and total electrons closely overlap, while the valence electrons significantly contribute only in low Q region. Since chemical bonds predominantly involve valence electrons, a refined approach employing only high-angle data (in the high Q region) effectively mitigates the effect of valence electrons, enabling the acquisition of accurate atomic positions and atomic displacement parameters.

Atomic scattering factors for the key constituents of Glycine ($C_2H_3NO_3$) and Cytidine ($C_5H_9N_2O_3$), namely C, N, and O elements, are depicted in Figure S1a through (c). For the structural analysis, only high-angle diffraction reflections ($0.70Å^{-1} \leq \sin\theta/\lambda$), in which core electrons dominate, were used to refine the structural parameters.

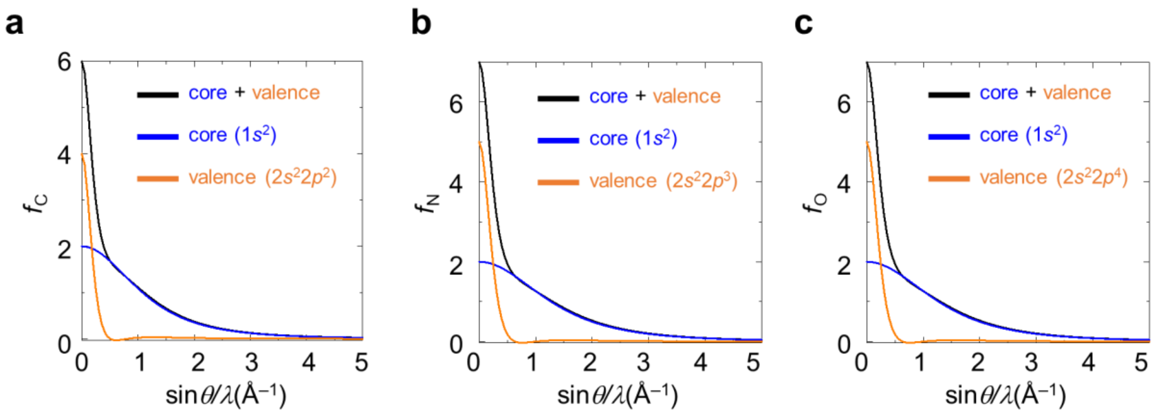

Figure S1. Atomic scattering factors of (a) C, (b) N, and (c) O elements.

The molecular and crystal structures of Glycine and Cytidine (**Figures S2-S3**) and the results of high-angle analysis (**Tables S1-S6**) are presented below.

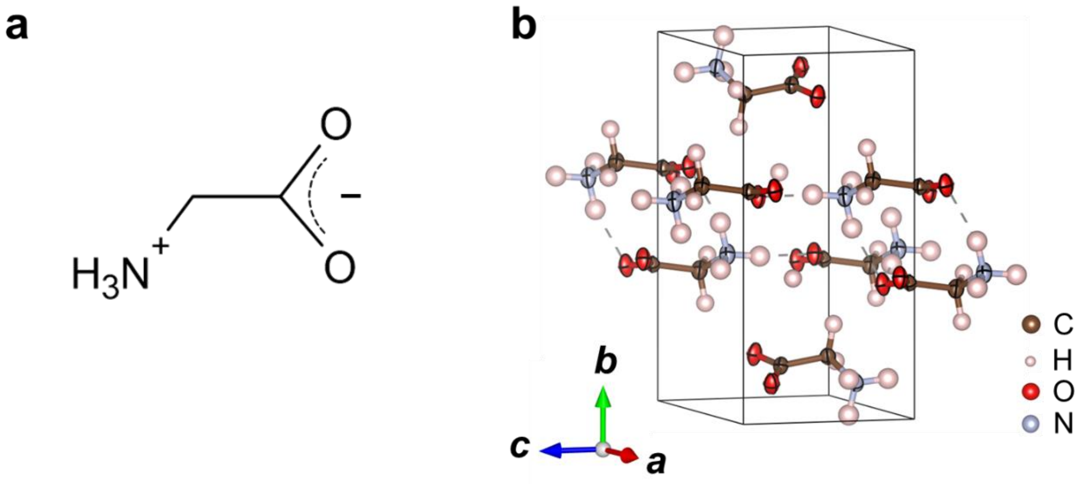

Figure S2. (a) Molecular structure and (b) crystal structure of Glycine. The gray dashed lines indicate hydrogen bonds. Atoms are shown by displacement ellipsoids (probability: 99%)

Table S1. Summary of crystallographic data of Glycine.

	Glycine
Chemical formula	$C_2H_3N_1O_2$
Temperature (K)	45
Wavelength (Å)	0.30920
Crystal dimension (μm^3)	170×70×70
Space group	$P2_1/n$
a (Å)	5.08950(10)
b (Å)	11.7893(2)
c (Å)	5.46580(10)
β (°)	112.023(8)
V (Å^3)	304.03(2)
Z	4

F (000)	160
$(\sin\theta / \lambda)_{\mathrm{Max}}$ (Å^{-1})	1.786
$N_{\mathrm{Total,\ obs}}$	150434
$N_{\mathrm{Unique,\ obs}}$	14218
Average redundancy	10.6
Completeness	0.965
R_1 ($I > 3\sigma$, 0.70Å^{-1} ≤ sinθ/λ ≤ 1.786Å^{-1}) [# of reflections]	0.0237 [9722]
GOF ($I > 3\sigma$, 0.70Å^{-1} ≤ sinθ/λ ≤ 1.786Å^{-1}) [# of reflections]	1.38 [9722]

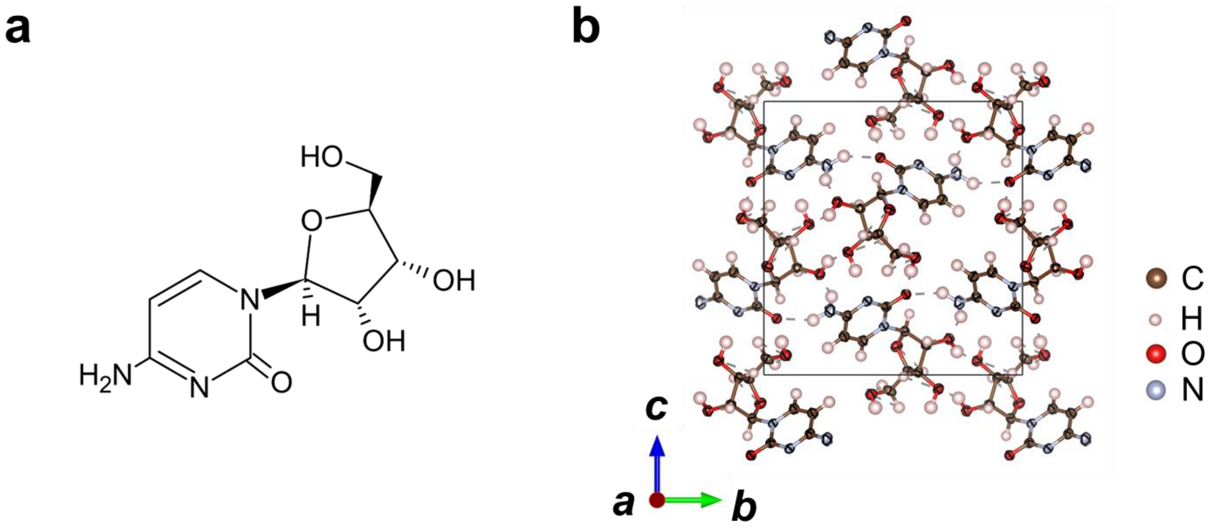

Figure S3. (a) Molecular structure and (b) crystal structure of Cytidine. The gray dashed lines indicate hydrogen bonds. Atoms are shown by displacement ellipsoids (probability: 99%)

Table S2. Summary of crystallographic data of Cytidine.

	Cytidine
Chemical formula	$C_9H_{13}N_3O_5$
Temperature (K)	35
Wavelength (Å)	0.33550
Crystal dimension (μm^3)	100×60× 60
Space group	$P2_12_12_1$
a (Å)	5.05220(10)
b (Å)	13.8915(3)
c (Å)	14.6782(3)
V (Å^3)	1030.15(4)
Z	4
F (000)	512
$(\sin\theta / \lambda)_{Max}$ (Å^{-1})	1.6565
$N_{Total, obs}$	206421
$N_{Unique, obs}$	37753
Average redundancy	5.5
Completeness	0.953
R_1 ($I > 3\sigma$, 0.70Å^{-1} ≤ $\sin\theta/\lambda$ ≤ 1.6565Å^{-1}) [# of reflections]	0.0258 [28150]
GOF ($I > 3\sigma$, 0.70Å^{-1} ≤ $\sin\theta/\lambda$ ≤ 1.6565Å^{-1}) [# of reflections]	1.08 [28150]

To assure data quality, attention should be paid to X-ray damage. The frame scale values in these experiments, which reflect the effect of X-ray damage, are shown in Figure S4. No indications of X-ray damage were found in these data.

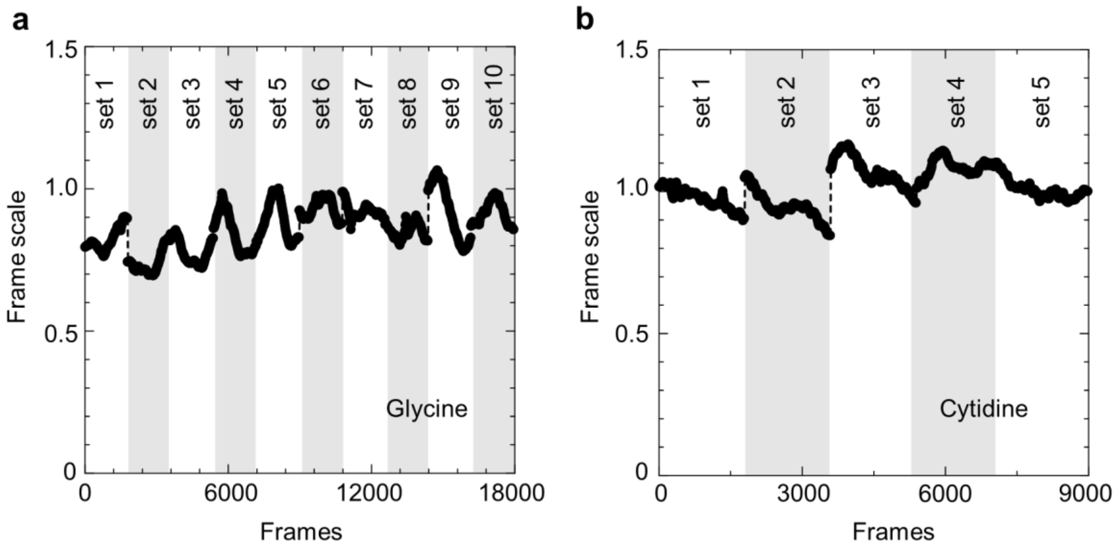

Figure S4. Frame scales of Glycine and Cytidine. Ten sets of single crystal XRD were performed under different rotation conditions for glycine and five sets for Cytidine. Each set (ω = 0 ~ 180 deg.) consists of 1800 frames, with a sweep angle $\Delta\omega$ of 0.1 degree per frame. The continuous change in scale values within each set is the effect of the shape factor due to the rotation of the crystals. Since the frame scales do not monotonically decrease throughout the measurements, it can be said that X-ray damages do not occur or were negligible for Glycine and Cytidine used in these experiments.

Supporting Section 2 | Straightforward model for describing the covalent bond derived from sp hybridized orbitals composed of 2s and 2p orbitals

In this section, we present the theoretically calculated the distribution along a carbon-carbon single bond, using sp hybridized orbitals composed of 2s and 2p orbitals. The hybridized orbital on the isolated carbon (C1) atom is expressed as $\phi_{C1}(\boldsymbol{r}_1) = \sqrt{\frac{1}{3}}\,\phi_{2s}(\boldsymbol{r}_1) - \sqrt{\frac{2}{3}}\,\phi_{2px}(\boldsymbol{r}_1)$, and its distribution is illustrated in Figure S5b, where $\phi_{2s}(\boldsymbol{r})$ and $\phi_{2px}(\boldsymbol{r})$ represents the 2s and $2p_x$ orbitals of the carbon atom, respectively. Radial distribution functions of $\phi_{2s}(\boldsymbol{r})$ and $\phi_{2px}(\boldsymbol{r})$ are calculated using the Hartree-Fock method [S10]. We initially consider a simple model in which the bonding orbitals between adjacent identical atoms are expressed as $\Psi(\boldsymbol{r}) = C(\phi_{C1}(\boldsymbol{r}) + \phi_{C2}(\boldsymbol{r}))$, and its distribution is illustrated at Figure S5 for bond length of $|\boldsymbol{r}_1 - \boldsymbol{r}_2| \sim 1.5$ Å, where C is the normalization constant. When compared to the experimental VED shown in Figures 1a and 1b, $|\Psi(\boldsymbol{r})|^2$ exhibits a relatively uniform distribution with a small dip at the center of the C1–C2 bond.

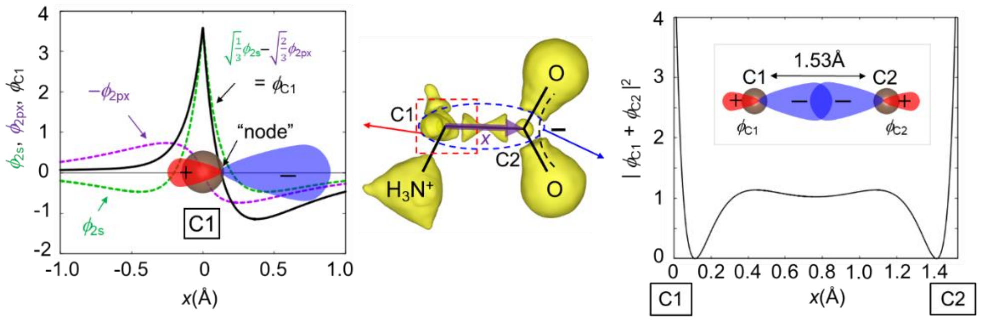

Figure S5. theoretically calculated the distribution along a carbon-carbon single bond, using sp hybridized orbitals composed of 2s and 2p orbitals. Central panel shows molecular structure and 3D surface map of experimental VED $\rho_{CDFS}(\boldsymbol{r})$ of a Glycine molecule, with the isosurface value set at $1.6e/\text{Å}^3$. Left panel shows the theoretical hybrid orbital $\phi_{C1}(\boldsymbol{r})$ consisting of 2s and 2p orbitals at the center of C1 atom. Right panel shows the calculated 1D plot illustrating the distribution of the bonding orbital $|\Psi(\boldsymbol{r})|^2$ (= $|\phi_{C1}(\boldsymbol{r}) + \phi_{C2}(\boldsymbol{r})|^2$) along the C1–C2 bond.

Supporting Section 3 | DFT calculation

In this section, we present the theoretically calculated canonical valence molecular orbitals of Glycine and Cytidine molecules. In our calculations, we used the long-range correction (LC) for Becke exchange and Lee-Yang-Parr correlation (BLYP) functional with the parameter value of μ=0.47, and employed the cc-pVTZ basis set in the GAMESS-US program. For the valence orbitals, it is important to note that LC-DFT, including LC-BLYP, has been verified to give accurate orbital energies and to closely approximate the orbital characteristics of the Dyson orbitals, which are essentially one-electron wavefunctions originating from high-level *ab initio* coupled-cluster wavefunctions. This alignment underscores the substantial accuracy of these valence orbitals.

When visualizing the π-bond from the experimental VED of Cytidine, we used $\rho_{\mathrm{DFT},2\sigma}(\boldsymbol{r})$ as the σ-bond contribution, subtracting the distribution of molecular orbitals corresponding to the π-orbitals (Figure S6) from the theoretical VED $\rho_{\mathrm{CDFS}}(\boldsymbol{r})$.

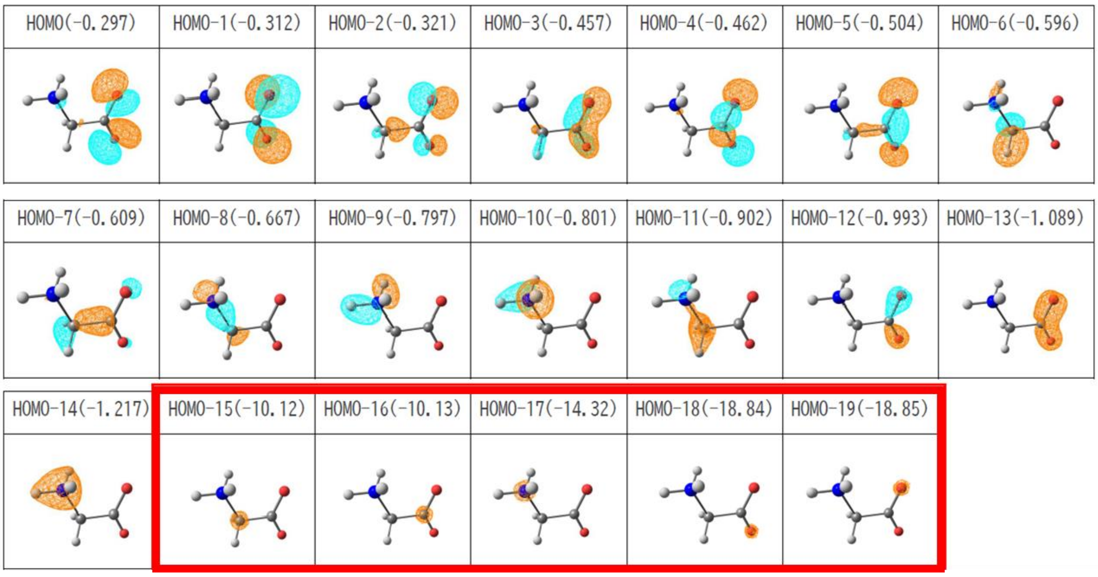

Figure S6. Canonical occupied molecular orbital (CMO) images and the corresponding orbital energies (in parentheses) of Glycine molecule, which are calculated using LC-BLYP/cc-pVTZ in GAMESS program. The CMOs in the red frame correspond to the core orbitals.

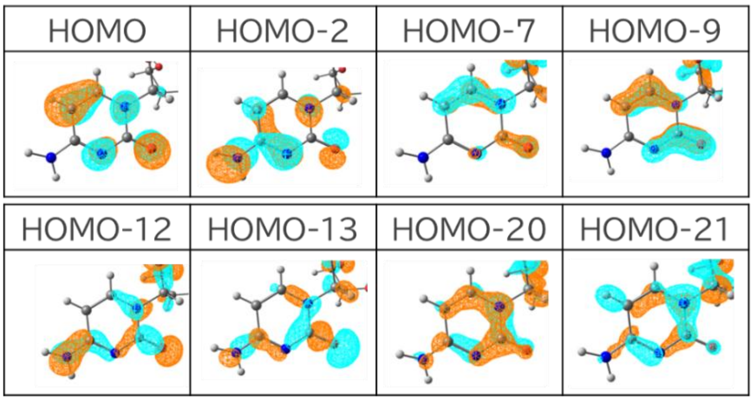

Figure S7. Canonical molecular orbital (CMO) images of Cytidine molecule for the valence occupied π-orbitals within the six-membered ring, which are calculated using LC-BLYP/cc-pVTZ in the GAMESS program. The CMO images are illustrated with the isosurface of 0.05 e/a.u.^{3}

Supporting Section 4 | Effects of spatial resolution and temperature on VED

In this section, we elucidate the interplay between the spatial resolution, temperature, and the resulting valence electron density distribution. Our experimental VEDs with a spatial resolution of 0.28 Å exhibit the fine structures, such as nodes and concavities (Figures 1a and 1b), whereas a previous study [S11] did not detect such a nodal plane in the total electron density distribution of Glycine molecules, which was based on laboratory X-ray diffraction data with a resolution of 0.45 Å. Therefore, we performed a Fourier transform using experimental data up to 0.5 Å, and the resulting Valence Electron Density (VED) is presented in Figure S8. Low-resolution experimental VED has a smoother distribution and exhibits no nodal plains or concavities. Consequently, there exists a distinct correlation between the spatial resolution of the data and the resultant VED distribution. To discuss the nature of chemical bonds, it is essential to acquire such high-resolution data, necessitating the utilization of high-quality, high-resolution X-rays obtained only at a synchrotron radiation facility.

Next, experiments on Cytidine were performed at two different temperatures, 35 K and 100 K. In general, higher temperatures lead to a reduction in diffraction reflection intensity at high angles, subsequently diminishing the spatial resolution. As a result, the spatial resolution of the data measured at 35 K is 0.30 Å, whereas the spatial resolution of the data measured at 100 K stands at 0.50 Å. Indeed, the divergence in spatial resolution becomes evident in the VED distribution, with the 35 K data exhibiting fine structures that are absent in the 100 K data (Figure S8b). Therefore, it is imperative to perform experiments employing high-energy X-ray at lower temperatures to ensure the acquisition of high-resolution data.

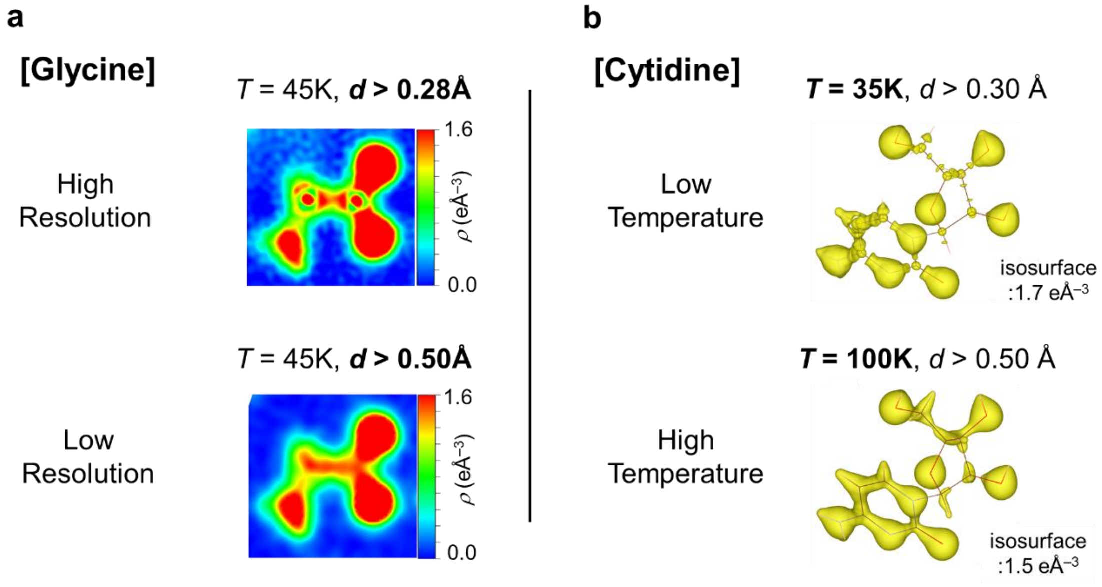

Figure S8. (a) 2D contour plots of experimental VED $\rho_{\mathrm{CDFS}}(\boldsymbol{r})$ for a Glycine molecule at high and low resolutions and (b) 3D surface plots of $\rho_{\mathrm{CDFS}}(\boldsymbol{r})$ for a Cytidine molecule at low and high temperatures.

Supporting Section 5 | The quantitative differences between $\rho_{CDFS}(\boldsymbol{r})$ and $\rho_{DFT}(\boldsymbol{r})$

In this section, we elucidate the factors responsible for the quantitative disparities between the experimental and calculated VEDs, as indicated in Figures 1b and 1d. Figure S9 presents a 1D curve plot tracing the C1–C2 bond of a Glycine molecule, while Figure S10 exhibits 1D curve plots for the C2–C1, C2–C3, and C4–C5 bonds of a Cytidine molecule. Note that the electron density observed along the bonds and just above the atom positions in $\rho_{DFT}(\boldsymbol{r})$ surpasses the corresponding experimental values for both the Glycine and Cytidine molecules.

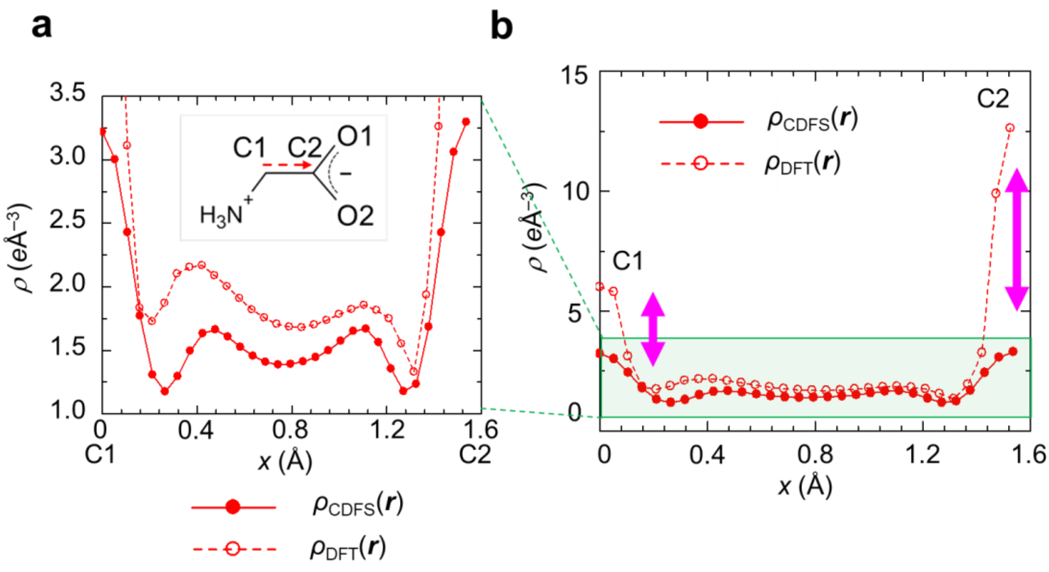

Figure S9. 1D-plot of $\rho_{CDFS}(\boldsymbol{r})$ and $\rho_{DFT}(\boldsymbol{r})$ on C1–C2 of Glycine.

((a): 1.0 eÅ^{-3} < $\rho(\boldsymbol{r})$ < 3.5 eÅ^{-3}, (b): -1 eÅ^{-3} < $\rho(\boldsymbol{r})$ < 15 eÅ^{-3})

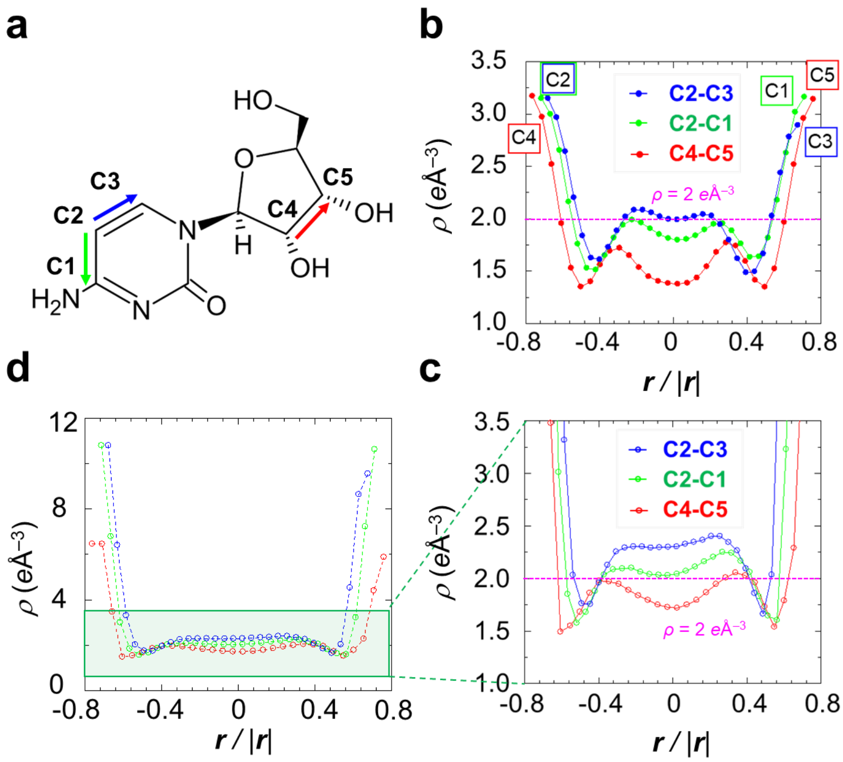

Figure S10. (a) Geometry of the paths in the following curve (1D) plots of Cytidine. The curve (1D) plots of (b) $\rho_{\mathrm{CDFS}}(\boldsymbol{r})$ and (c), (d) $\rho_{\mathrm{DFT}}(\boldsymbol{r})$ on C2–C1, C2–C3, C4–C5. (c): 1.0 eÅ^{-3} < $\rho(\boldsymbol{r})$ < 3.5 eÅ^{-3}, (d): –1 eÅ^{-3} < $\rho(\boldsymbol{r})$ < 12 eÅ^{-3}).

The quantitative disparities can be attributed to the differing approaches taken in managing temperature effects between experiments and theoretical data. While LC-DFT calculations are carried out for the ground state at 0 K, the experiments are conducted at finite temperatures. To comprehensively investigate these temperature effects, we performed a rigorous validation exercise using Cytidine data. In the following verifications and comparisons, the core electron density $\rho_{\mathrm{CDFS}}^{core}(\boldsymbol{r})$ employed by the CDSF method is derived from the atomic scattering factors. In contrast, the LC-DFT calculations uses $\rho_{\mathrm{DFT}}^{core}(\boldsymbol{r})$, representing to the electron density distribution of the core electron orbitals.

In the crystal structure analysis, the temperature effect is incorporated in the following tabular expression.

$$F_C(\boldsymbol{K}) = \sum_{\boldsymbol{j}} f_j T_j(\boldsymbol{K}) \exp(-2\pi i \boldsymbol{K} \cdot \boldsymbol{r}) \qquad \cdots (S1)$$

$$T_j(\boldsymbol{K}) = \exp[-2\pi^2\{h^2(a^*)^2U_{11} + k^2(b^*)^2U_{22} + l^2(c^*)^2U_{33} + 2hk(a^*b^*)^2U_{12} + 2kl(b^*c^*)^2U_{23} + 2lh(c^*a^*)^2U_{31}\}] \quad \cdots (S2)$$

The anisotropic temperature factors U_{11} ~ U_{31} are values refined through crystallographic analysis. However, these factors cannot be directly incorporated into the theoretical electron density distribution. We consequently introduce $s(\boldsymbol{K})$ (= $F^{core}_{\mathrm{CDFS,35K}}(\boldsymbol{K})/F^{core}_{\mathrm{CDFS,0K}}(\boldsymbol{K})$), signifying the ratio of the refined crystal structure factor of core electrons $F^{core}_{\mathrm{CDFS,35K}}(\boldsymbol{K})$, wherein the temperature factor is considered, and $F^{core}_{\mathrm{CDFS,0K}}(\boldsymbol{K})$ (U_{11} ~ U_{31} = 0), where the temperature factor is not considered. This parameter $s(\boldsymbol{K})$ means the temperature effect for each reflection. Subsequently, we derive $\rho^{core}_{\mathrm{DFT}}(\boldsymbol{r})$ that accounts for temperature effects, denoted as $\rho^{core}_{\mathrm{DFT},35K}(\boldsymbol{r})$, by performing an inverse Fourier transform of $F^{core}_{\mathrm{DFT,35K}}(\boldsymbol{K}) \coloneqq s(\boldsymbol{K}) \cdot F^{core}_{\mathrm{DFT}}(\boldsymbol{K})$, where $F^{core}_{\mathrm{DFT}}(\boldsymbol{K})$ represents the Fourier transform of $\rho^{core}_{\mathrm{DFT}}(\boldsymbol{r})$.

Figure S11 shows the double logarithmic plots of (a) $\left|F^{core}_{\mathrm{CDFS,35K}}(\boldsymbol{K})\right|^2$ - $|F^{core}_{\mathrm{DFT}}(\boldsymbol{K})|^2$ and (b) $\left|F^{core}_{\mathrm{CDFS,35K}}(\boldsymbol{K})\right|^2$ and $\left|F^{core}_{\mathrm{DFT,35K}}(\boldsymbol{K})\right|^2$. Note that a systematic and substantial discrepancy is found between $\left|F^{core}_{\mathrm{CDFS,35K}}(\boldsymbol{K})\right|^2$ and $|F^{core}_{\mathrm{DFT}}(\boldsymbol{K})|^2$. Conversely, $\left|F^{core}_{\mathrm{DFT,35K}}(\boldsymbol{K})\right|^2$ exhibits a remarkable agreement with $\left|F^{core}_{\mathrm{CDFS,35K}}(\boldsymbol{K})\right|^2$. Furthermore, Figure S12 presents the 1D plots of $\rho^{core}_{\mathrm{CDFS}}(\boldsymbol{r})$, $\rho^{core}_{\mathrm{DFT}}(\boldsymbol{r})$, $\rho^{core}_{\mathrm{DFT,35K}}(\boldsymbol{r})$ on C2–C1 in a Cytidine molecule. Note that $\rho^{core}_{\mathrm{DFT,35K}}(\boldsymbol{r})$ exhibits a clear and consistent agreement with $\rho^{core}_{\mathrm{CDFS}}(\boldsymbol{r})$. As discussed in the main article, qualitative comparisons can be conducted without the aforementioned procedure. However, for precise and quantitative comparisons between experiments and theoretical results, meticulous attention to handling temperature effects is required.

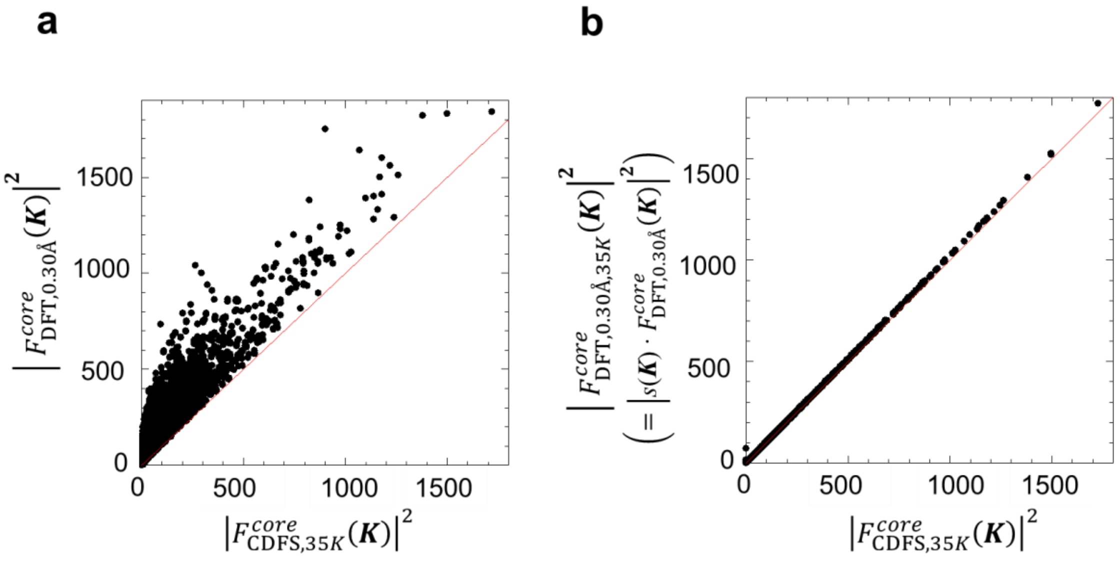

Figure S11. Double logarithmic plots of (a) $|F^{core}_{\mathrm{CDFS,35K}}(\boldsymbol{K})|^2 - |F^{core}_{\mathrm{DFT,0.30Å}}(\boldsymbol{K})|^2$ and (b) $|F^{core}_{\mathrm{CDFS,35K}}(\boldsymbol{K})|^2 - |F^{core}_{\mathrm{DFT,0.30Å,35K}}(\boldsymbol{K})|^2$.

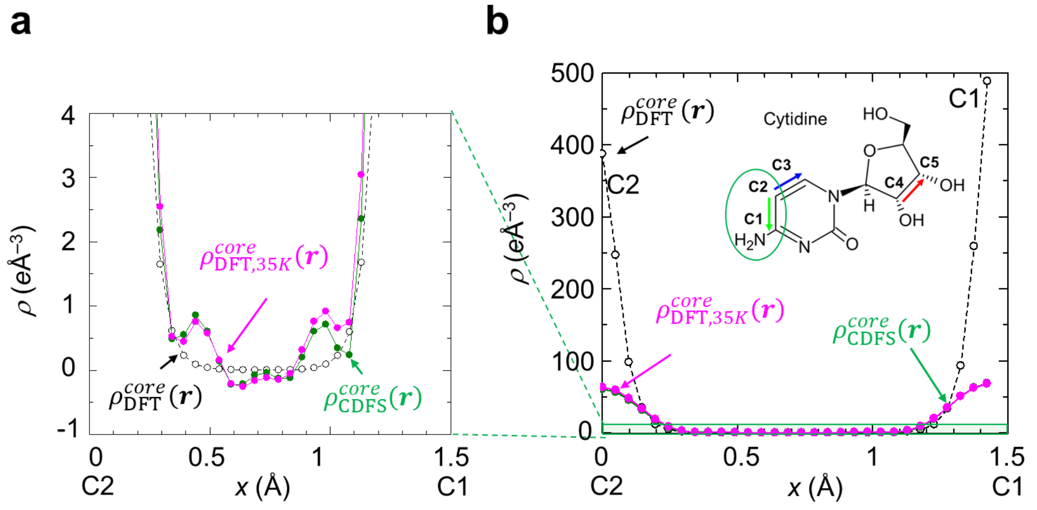

Figure S12. The curve (1D) plots of $\rho^{core}_{\mathrm{CDFS}}(\boldsymbol{r})$, $\rho^{core}_{\mathrm{DFT}}(\boldsymbol{r})$, and $\rho^{core}_{\mathrm{DFT,35K}}(\boldsymbol{r})$ on C1–C2 of Cytidine. ((a) –1 eÅ^{-3} < $\rho(\boldsymbol{r})$ < 4 eÅ^{-3}, (b) –1 eÅ^{-3} < $\rho(\boldsymbol{r})$ < 500 eÅ^{-3}).

Supporting Section 6 | The experimental VEDs of C–O, C–N and C–H bonds in a Glycine molecule

The 1D curve plots of experimental and theoretical VEDs on a C–O, C–N, and C–H bonds in a Glycine molecule are shown in Figures S13-S15. Unlike the C–C covalent bonds shown in Figure 1, these bonds, composed of different atoms, showed an asymmetric distribution, reflecting differences in electronegativity.

Figure S13a provides a 1D plot of $\rho_{\mathrm{CDFS}}(\boldsymbol{r})$ on the C2–O1 and C2–O2 bonds in a Glycine molecule. Considering the bond lengths and VEDs, there hardly is difference between the C2–O1 and C2–O2 bonds, which is also confirmed by the theoretical VED $\rho_{\mathrm{DFT}}(\boldsymbol{r})$ in Figure S13b. These results indicate that the electrons are delocalized over O1 and O2, forming nearly identical C2–O1 and C2–O2 bonds through resonance, as is typical in the stabilization of the carboxylate ions.

In the two C–H bonds in a Glycine molecule, a qualitative difference between experimental and theoretical VED is also evident. Figure 15a illustrates the 1D plots on the C1–H4 and C1–H5. In experimental VED, the electron density of H5 atom is higher than that of H4 atom, whereas in theoretical VED, the electron densities of H4 and H5 atoms are almost identical. In the crystal, H5 atom exists within the two-dimensional hydrogen bonding network in the *ac* plane. It approaches a charge-neutral state to reduce Coulombic repulsion, suggesting that H5 atom possesses more electrons.

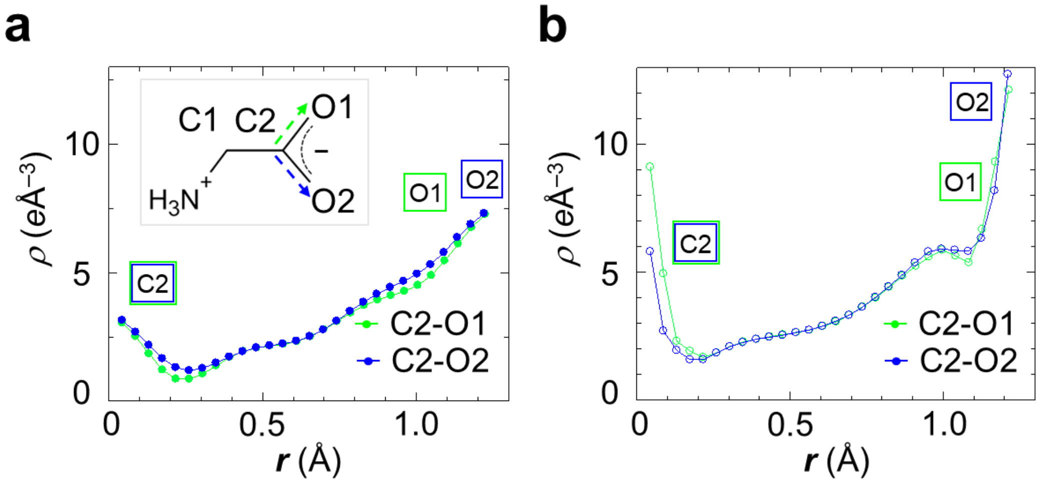

Figure S13. The curve (1D) plots of (a) experimental CDFS VED $\rho_{\mathrm{CDFS}}(\boldsymbol{r})$ and (b) theoretical DFT VED $\rho_{\mathrm{DFT}}(\boldsymbol{r})$ of Glycine on C2–O1 and C2–O2 bonds. The values at the just atoms are excluded. The experimental bond lengths of the C2–O1 and C2–O2 bonds are 1.26052(11) Å and 1.25748(14) Å, respectively.

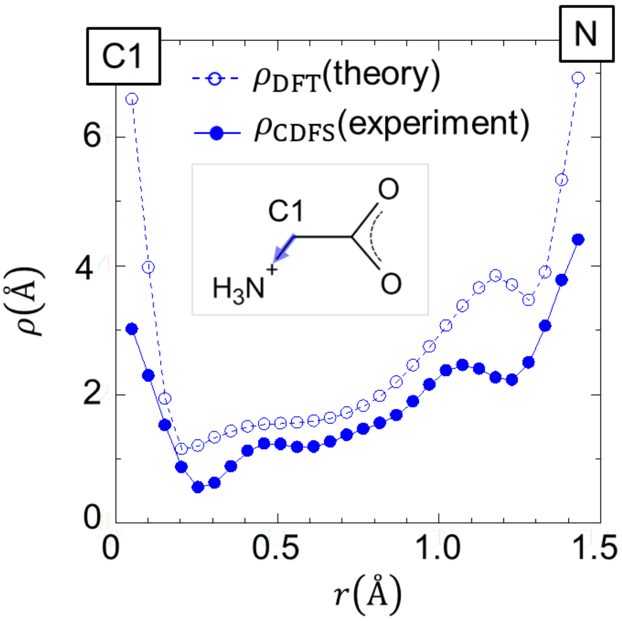

Figure S14. The curve (1D) plots of experimental CDFS VED $\rho_{\mathrm{CDFS}}(\boldsymbol{r})$ and theoretical DFT VED $\rho_{\mathrm{DFT}}(\boldsymbol{r})$ of Glycine on the C1–N bonds. The values at the just atoms are excluded.

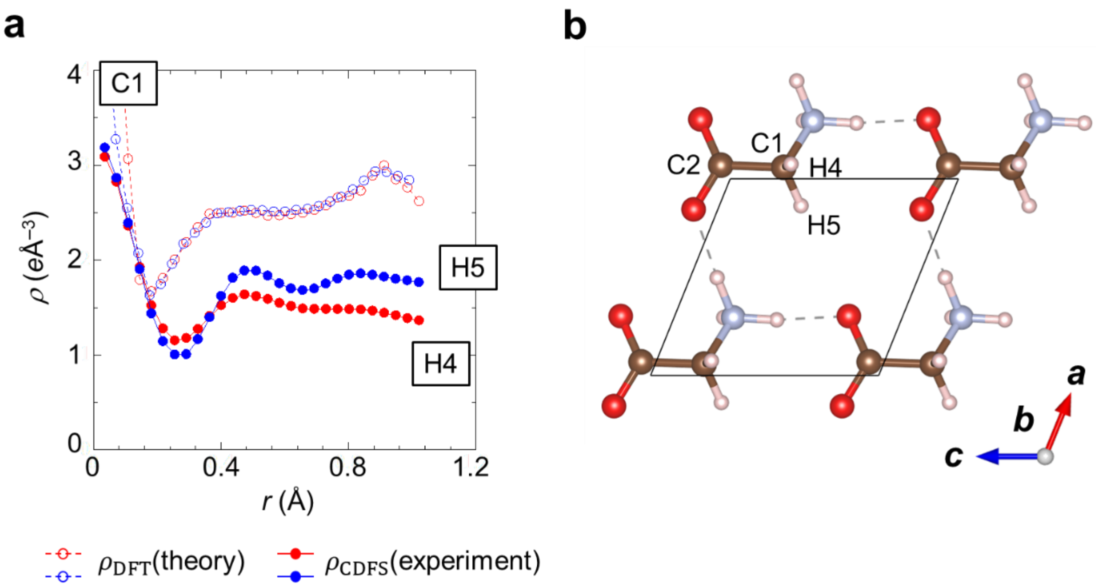

Figure S15. (a) The curve (1D) plots of experimental CDFS VED $\rho_{\mathrm{CDFS}}(\boldsymbol{r})$ and theoretical DFT VED $\rho_{\mathrm{DFT}}(\boldsymbol{r})$ of Glycine on the C1–H4 and C1–H5 bonds. The values at the just atoms are excluded. (b), The crystal structure of Glycine in an *ac* plain.

Supporting Section 7 | Visualizing the distribution of π-bond from experimental VEDs subtracting the theoretical distribution of σ–bond

Figure S16 provides the process of visualization for π-orbital distribution in C1–C2 bond. Contrary to expectations, the theoretical 2sσ distribution of the C1–C2 bond, $\rho_{\mathrm{DFT},2\sigma}(\boldsymbol{r})$, shows a spread in the molecular plane direction rather than perfect axial symmetry. This is thought to be due to the overlapping contribution of the electrons in the σ-bond between C2 atom and the adjacent atoms other than C1 and the Coulomb repulsion with the π electrons. Therefore, the nearly axisymmetric distribution of $\rho_{\mathrm{CDFS}}(\boldsymbol{r})$ with the contributions from σ– and π– orbitals, is reasonable.

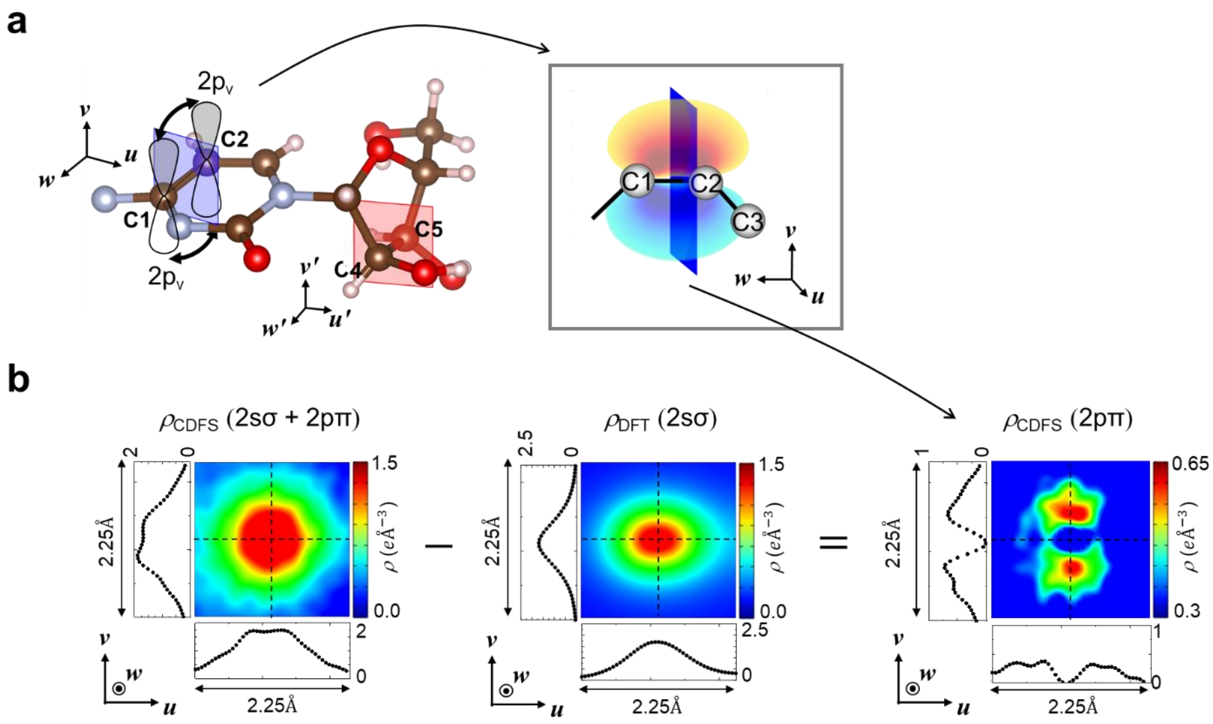

Figure S16. (a) Locations of the cross sections in Cytidine molecule for the following 2D contour plots. The locations of the bond cross sections of C1–C2 and C4–C5 are represented by the blue plane and the red plane, respectively. The locations of these planes are in the quarter of the vector from C2 to C1. The picture in the gray box depicts the relationship between the blue plane and the distribution of π–orbital on C1–C2. (b) The process of visualization for π-orbital distribution in C1–C2 bond represented by 2D contour plots of the bond cross sections of $\rho_{CDFS}(\boldsymbol{r})$, $\rho_{DFT,2\sigma}(\boldsymbol{r})$, and $\rho_{CDFS}(\boldsymbol{r})-\rho_{DFT,2\sigma}(\boldsymbol{r})$.

Supporting References

[S1] Dawson, B. Aspherical Atomic Scattering Factors in Crystal Structure Refinement I. Coordinate and Thermal Motion Effects in a Model Centrosymmetric System. *Acta Cryst.* **1964**, *17*, 990–996.

[S2] Stewart, R. F. Valence Structure from Coherent X-Ray Scattering: Fourier Difference Synthesis. *J. Chem. Phys*. **1968**, *48*, 4882–4889.

[S3] Stewart, R. F. On the Dependence of X-Ray Debye-Waller Parameters on Atomic Form Factors. *Acta Cryst. A* **1973**, *29*, 602–605.

[S4] Stewart, R. F. Electron Population Analysis with Generalized X-Ray Scattering Factors: Higher Multipoles. *J. Chem. Phys.* **1973**, *58*, 1668–1686.

[S5] Sakurai,T., Ito, T. On the Effect of Asphericity of the Atomic Scattering Factor on the Temperature Factor. *Acta Cryst. B* **1969**, *25*, 1031–1038.

[S6] Groenewegen, P. P. M.; Zeevalkink, J.; Feil, D. X-Ray Scattering and the Chemical Bond in N_2 and CN–. *Acta Cryst*. *A* **1971**, *27*, 487–491.

[S7] Ruysink, A. F. J.; Vos, A. The Structures of Crystals Containing *trans* and *cis* Molecules of 2,5-Dimethyl-3-hexene-2,5-diol, $C_8H_{16}O_2$, at –160°C. *Acta Cryst*. *B* **1974**, *30*, 1997–2002.

[S8] Jeffrey, G. A.; Cruickshank, D. W. J. Molecular Structure Determination by X-Ray Crystal Analysis: Modern Methods and Their Accuracy. *Quart. Rev. Chem. Soc. Lond.* **1953**, *7*, 335–376.

[S9] Bentley, J.; Stewart, R. F. Core Deformation Studies by Coherent X-ray Scattering. *Acta Cryst. A* **1974**, *30*, 60–67.

[S10] Su, Z., Coppens, P. Nonlinear Least-Squares Fitting of Numerical Relativistic Atomic Wave Functions by a Linear Combination of Slater-Type Functions for Atoms with Z = 1–36. *Acta Cryst. A* **1998**, *54*, 646–652.

[S11] Destro, R., Roversi, P., Barzaghi, M. & Marsh, R. E. Experimental Charge Density of α-Glycine at 23 K. *J. Phys. Chem. A* **2000**, *104*, 1047–1054.
